# The Holistic Coronary Physiology Display: Calculation of the Flow Separation Index in Vessel-Specific Individual Flow Range during Fractional Flow Reserve Measurement Using 3D Coronary Reconstruction

**DOI:** 10.3390/jcm10091910

**Published:** 2021-04-28

**Authors:** Gábor Tamás Szabó, Áron Üveges, Balázs Tar, András Ágoston, Azzaya Dorj, Csaba Jenei, Rudolf Kolozsvári, Benjamin Csippa, Dániel Czuriga, Zsolt Kőszegi

**Affiliations:** 1 Division of Cardiology, Department of Cardiology, Faculty of Medicine, University of Debrecen, Móricz Zs. krt. 22, 4032 Debrecen, Hungary; nszgt@med.unideb.hu (G.T.S.); azaad517@gmail.com (A.D.); csjenei@med.unideb.hu (C.J.); kolozsvari.rudolf@med.unideb.hu (R.K.); dczuriga@med.unideb.hu (D.C.); 2 Kálmán Laki Doctoral School of Biomedical and Clinical Sciences, University of Debrecen, 4032 Debrecen, Hungary; aronaok@gmail.com (Á.Ü.); tarbalazsdr@gmail.com (B.T.); agostonandras48@gmail.com (A.Á.); 3 Szabolcs–Szatmár–Bereg County Hospitals and University Teaching Hospital, 4400 Nyíregyháza, Hungary; 4 Department of Hydrodynamic Systems, Budapest University of Technology and Economics, 1111 Budapest, Hungary; csippa.benjamin@gmail.com

**Keywords:** coronary flow reserve, fractional flow reserve, 3D reconstruction

## Abstract

In order to make optimal decisions on the treatment of atherosclerotic coronary heart disease (CHD), appropriate evaluation is necessary, including both the anatomical and physiological assessment of the coronary arteries. According to current guidelines, a fractional flow reserve (FFR)–based clinical decision is recommended, but coronary flow reserve (CFR) measurements and microvascular evaluation should also be considered in special cases for a detailed exploration of the coronary disease state. We aimed to generate an extended physiological evaluation during routine FFR measurement and define a new pathological flow–related prognostic factor. Fluid dynamic equations were applied to calculate CFR on the basis of the three-dimensional (3D) reconstruction of the invasively acquired coronary angiogram and the measured intracoronary pressure data. A new, potentially robust prognostic parameter of a coronary lesion called the “flow separation index” (FS_i_), which is thought to detect the pathological flow amount through a stenosis was introduced in a vessel-specific flow range. Correlations between FS_i_ and the clinically established physiological indices (CFR and FFR) were determined. The FS_i_ was calculated in 19 vessels of 16 patients, including data from the pre- and post-stent revascularization treatment of 3 patients. There was no significant correlation between the FS_i_ and the CFR (r = −0.23, *p* = 0.34); however, there was significant negative correlation between the FS_i_ and the FFR (r = −0.66, *p* = 0.002). An even stronger correlation was found between the FS_i_ and the ratio of the resting pressure ratio and the FFR (r = 0.92, *p* < 0.0001). The diagnostic power of the FS_i_ for predicting the FFR value of <0.80, as a gold standard prognostic factor, was tested by receiver operating characteristic analysis. FS_i_ > 0.022 proved to be the cutoff value of the prediction of a pathologically low FFR with a 0.856 area under the curve (95% confidence interval: 0.620 to 0.972). The present flow–pressure–velocity display provides a comprehensive summary of patient-specific pathophysiology in CHD. The consequences of epicardial stenoses can be evaluated together with their complex relations to microvascular conditions. Based on these values, clinical decision-making concerning both pharmacological therapy and percutaneous or surgical revascularization may be more precisely guided.

## 1. Introduction

For optimal decision-making in the treatment of atherosclerotic coronary heart disease (CHD) including pharmacotherapy, as well as percutaneous coronary interventions (PCI) or surgical treatment, the adequate evaluation of coronary angiographic results with quantitative anatomical and physiological assessments is recommended [1].

According to current guidelines, in the case of intermediate stenosis without objective evidence of ischemia, the measurement of the fractional flow reserve (FFR) is preferred during invasive coronary angiography [2,3]. FFR expresses the ratio between the mean distal coronary artery pressure measured by a pressure wire and the mean aortic pressure detected at the tip of the guiding catheter during pharmacologically induced maximal vasodilation [4]. Based on current clinical guidelines, an FFR value equal to or less than 0.80 is an indication for PCI [2,3]. However, in the Fractional Flow Reserve versus Angiography for Multivessel Evaluation 2 (FAME 2) study, patients with an FFR value above the cutoff ratio (0.80) also demonstrated a significant risk of adverse cardiovascular events, and 12% of these study participants required PCI. On the other hand, 52.6% of the patients randomized to medical therapy under the cutoff FFR value did not suffer an event during the 2-year follow-up period [5]. These data indicate the need for a more specific prognostic parameter than FFR.

A coronary lesion can also be evaluated by coronary flow reserve (CFR), which is defined as the ratio of the coronary flow (velocity) during maximal vasodilation to that in a resting state [6]. Hemodynamically significant coronary lesions are known to reduce CFR to as low as <2. In the case of diffuse coronary artery atherosclerosis, CFR can also be decreased to a pathological level; furthermore, an impaired CFR may indicate microvascular dysfunction.

In patients with suspected CHD, CFR proved to be an independent factor in terms of prediction of mortality [7]. The quantitative relation between the values of CFR and prognosis has also been established, providing meaningful incremental risk stratification [8].

In contrast with FFR, which represents the hemodynamic consequences of the epicardial resistance, CFR reflects the flow capacity of the entire coronary artery system, including the microcirculation [9]. Moreover, as FFR and CFR utilize entirely different approaches to describe coronary pathophysiology, pronounced discrepancies may occur between these two parameters [10]. This fact highlights the need for the incorporation of both pressure and flow parameters when aiming to describe lesion physiology [11].

Several attempts have been made to perform the simultaneous measurement of pressure-derived FFR and CFR using pressure measurements only [12,13,14,15,16]. However, given the systematic underestimation of CFR values [14], it has been concluded that CFR cannot be measured merely by pressure alone, as friction losses are not negligible across a native coronary artery stenosis, and the ratio of the flow separation resistance and the resistance from the laminar flow cannot be predicted from pressure data [13,14].

Recent publications proposed the “pressure-bounded” CFR (CFR_pb_) assessment for delineating the possible range of the CFR according to the measured resting and hyperemic pressures [15,16]. However, with the classification of the lesions into three distinct CFR_pb_ groups in retrospective studies, the approach has failed to specify whether CFR is in a normal or abnormal range in approximately half of the lesions [15,16].

A three-dimensional (3D) coronary tree reconstruction can be performed out of standard orthogonal two-dimensional (2D) images. Lesion characteristics show limitations with 2D quantitative coronary angiography [17], while the comparison of 2D vs. 3D coronary evaluations has demonstrated the superiority of 3D analysis in the detection of the functional severity of a lesion [18]. Another advantage of 3D reconstruction is related to the possibility of optimal flow modeling in the coronary arteries of interest.

Here, we aimed to introduce a hemodynamic calculation method by setting up a model, where 3D angiographic reconstruction was also incorporated. Previous evaluations have demonstrated the superiority of 3D vs. 2D analysis in the detection of the functional severity of a lesion. Moreover, 3D reconstruction has the advantage of optimal flow modeling in the coronary arteries of interest. We made efforts to augment the results of simultaneous intracoronary pressure measurement by exploiting the 3D analysis–related flow assessment during both vasodilation and the resting state, expressing the classic physiological parameter of CFR.

We established the flow separation index (FS_i_) as a new evaluation parameter that can also take into account the pathological turbulent flow in connection with low and oscillating shear stress, which play an important role in the progression of an atherosclerotic plaque [19,20,21]. Therefore, FS_i_ may potentially become a prognostic factor for the progression of CHD. Our main goal here was to explore the correlations between FS_i_ and the already established prognostic factors, FFR and CFR.

## 2. Materials and Methods

**Figure 1:**
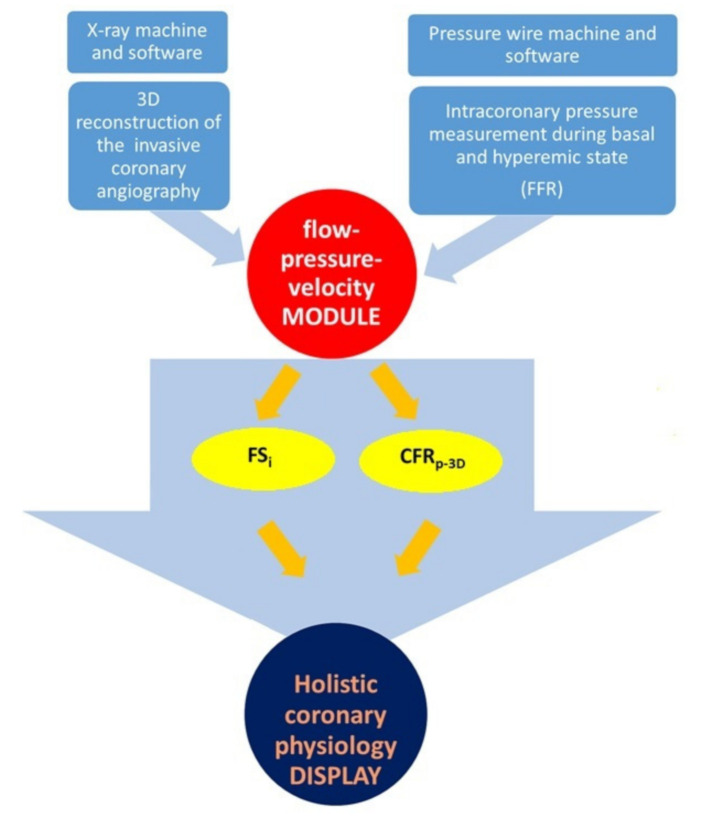
Flowchart representing the generation of the holistic coronary physiology display. Appropriate DICOM-formatted images were exported to the 3D reconstruction software, and the 3D parameters required for the flow calculations (A_p_, A_s_, A_d_, L_p,_ L_s_, L_d_, A_d_, and MLA) were provided automatically by the software (top left part of the figure). Intracoronary pressure data from the FFR measurement (top right part of the figure) were applied to the module (red circle in the middle) to calculate the basal and hyperemic flows and their ratio (CFR_p-3D_) as well as to generate the pressure–flow relation by quantifying the flow separation index (FS_i_) in the vessel-specific flow range. 3D: three dimensional; FFR: fractional flow reserve; FS_i_: flow separation index; CFR_p-3D_: 3D derived coronary flow resistance; DICOM: Digital Imaging and COmmunications in Medicine; A_p_: proximal reference area; A_s_: average stenotic area; A_d_: distal reference area; L_p_: length of the proximal reference area; L_s_: length of the stenotic area; L_d_: length of the distal reference area; A_d_: post-stenotic mean lumen diameter; MLA: minimum lumen area.

In our model, one of the stipulations was to avoid the use of Doppler or thermodilution methods, which can be technically challenging [10,22,23,24]. Therefore, our new calculation method uses data only from the routinely performed intracoronary pressure measurement and 3D parameters to assess pressure–flow relations [11]. Our model describes the flow–pressure relation. The flow chart of generating the holistic coronary physiology display is depicted in Figure 1.

### 2.1. Coronary Angiography and Fractional Flow Reserve Measurement

Diagnostic coronary angiography was performed and recorded with an AXIOM Artis-X-Ray device (Siemens, Munich, Germany), using standard fluoroscopic views. FFR measurement was performed with the RadiAnalyzer^TM^ device (St. Jude Medical, Saint Paul, MN, USA) and a pressure sensor guidewire PressureWire^TM^ Certus^TM^ (St. Jude Medical). The wire was calibrated before use, and pressures were equalized at the tip of the guiding catheter. The resting pressure curve was recorded after intracoronary (ic.) administration of glyceryl trinitrate and a flush of saline. Maximal vasodilation was reached with the ic. administration of 200 μg adenosine. For a distal wire position, the pressure sensor was introduced at least 20 mm below the coronary stenosis.

### 2.2. Three-Dimensional Quantitative Coronary Reconstruction

For the calculation of CFR_p-3D_, 3D angiographic reconstruction was performed following invasive coronary angiography with a dedicated, commercially available software (QAngio XA Research Edition 1.0, Medis Specials bv, Leiden). For the reconstruction, two angiographic recordings of sufficient visual quality are necessary, with at least a 25° difference between the projections. We modeled the interrogated vessel segment in 3D, from the coronary orifice to the level of the pressure wire sensor. In our display model, 3D imaging for acquiring highly accurate anatomical data was required. CFR_p-3D_ and FS_i_ values were calculated from the corresponding data of the precise anatomical reconstruction and intracoronary pressure measurement.

### 2.3. Calculation of CFR_p-3D_

**Figure 2:**
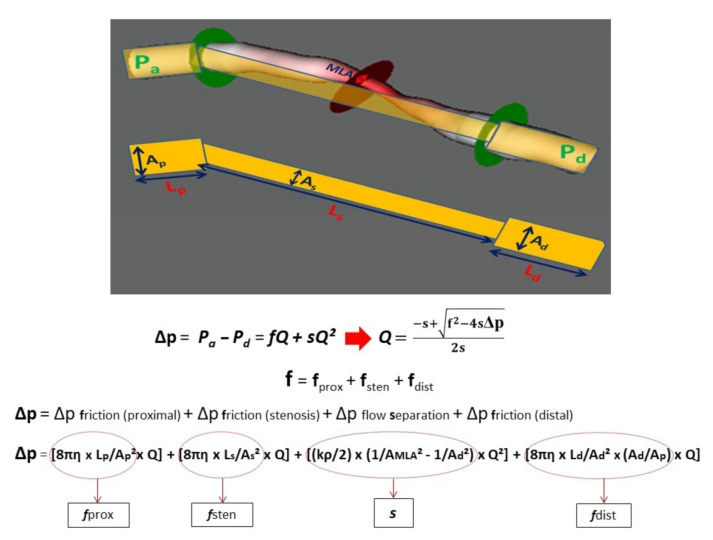
The calculation model of CFR_p-3D_. Simplified model consisting of three segments (proximal, stenotic, and distal) of the target vessel on the basis of 3D quantitative coronary angiography. The pressure drops were described according to classic fluid dynamic equations [25]. By using the measured pressure data, the equations were solved to calculate the flow. Pa: pressure in the proximal reference area; MLA: minimum lumen area; P_d_: pressure distal to the stenosis; A_p_: proximal reference area; A_s_: average stenotic area; A_d_: distal reference area; L_p_: length of the proximal reference area; L_s_: length of the stenotic area; L_d_: length of the distal reference area; Δp: total pressure drop along the target vessel; Q: volumetric flow; f_:_ linear coefficient in the viscous friction pressure loss; s: quadratic coefficient in the separation-related pressure loss term; η: blood viscosity; ρ: blood density.

The calculation model of CFR_p-3D_ is depicted in Figure 2. Classic fluid dynamic equations were used for the calculation of the flow–pressure function, as published previously [25]. According to this model, the total pressure difference (Δp), the linear coefficient of the viscous friction (f), and the quadratic term representing separation losses (s) as well as the volumetric flow value were determined. By calculating the flow rate in hyperemic and resting states, the CFR_p-3D_ value was computed as the ratio of the two flow rates.

The calculated CFR_p-3D_ as a classic index of functional coronary impairment was projected to display the pressure–flow relation of coronary circulation, providing information on the patient-specific pathophysiology. The scheme is a conceptually modified version of what Papafaklis et al. proposed as a virtual functional assessment display [26]. Their approach displayed the computed ratio of distal to aortic pressure (P_d_/P_a_) − Q relationship in a function of a hypothesized fixed flow range (0–4 mL/s).

**Figure 3:**
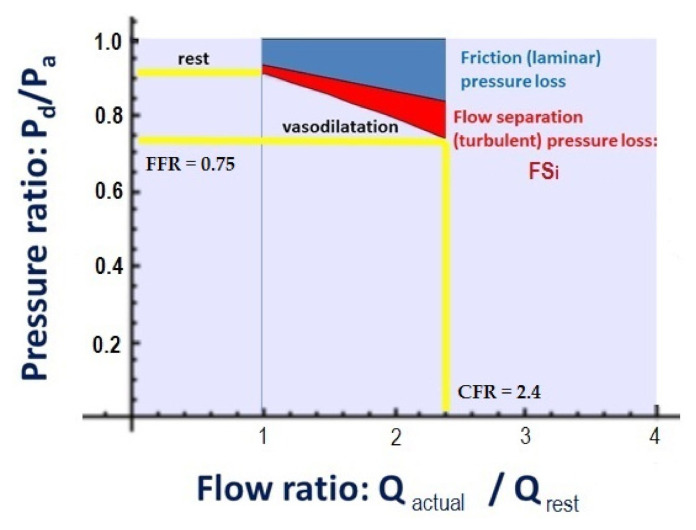
Definition of flow–pressure relation and FS_i_. The X axis represents the actual flow ratio to the resting flow, while the Y axis shows the distal to proximal (aortic) pressure ratio. FFR: fractional flow reserve; P_a_: pressure in the proximal reference area; P_d_: pressure distal to the stenosis; CFR: coronary flow reserve; FS_i_: flow separation index; Q_actual_: volumetric flow at actual state; Q_rest_: volumetric flow at resting state.

As opposed to this, in our scheme, the X axis represents the actual flow ratio to the resting flow, while the Y axis shows the distal to proximal (aortic) pressure ratio. Thus, the flow ratio can be read on the X axis, while the resting pressure ratio is shown on the Y axis and, at maximal hyperemia, the flow ratio shows the CFR, and the pressure ratio represents the FFR (Figure 3).

**Figure 4:**
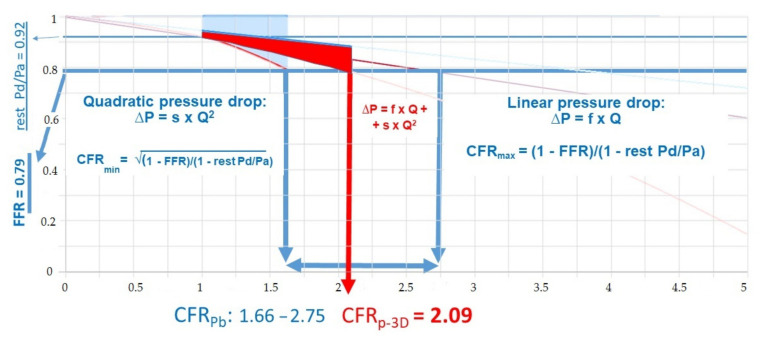
Relation of the pressure-bounded CFR (CFR_Pb_) interval and CFR_p-3D_. The X axis represents the actual flow ratio to the resting flow, while the Y axis shows the distal to proximal (aortic) pressure ratio. FFR: fractional flow reserve; P_a_: pressure in the proximal reference area; P_d_: pressure distal to the stenosis; Q: volumetric flow; s: quadratic coefficient in the separation-related pressure loss term; Δp: total pressure drop along the target vessel; CFR: coronary flow resistance; f: linear coefficient in the viscous friction pressure loss; CFR_Pb_: pressure-bounded coronary flow resistance; CFRp-3D: 3D derived coronary flow resistance; rest Pd/Pa: fractional flow reserve in the resting state; CFRmin: minimal value of the CFR; CFRmax: maximal value of the CFR.

The calculated CFR_p-3D_ always defines the exact CFR value within the pressure-bounded CFR interval, as depicted in Figure 4 [15,16].

Using only intracoronary pressure data, the lower and higher bounds of the CFR_Pb_ interval can be calculated by assuming the minimal CFR, as this would be the case with only a quadratic pressure drop, and the higher bound is defined as the maximal CFR, as this would be the case with only a linear pressure drop. The exact combination of the two types of flow resistances can be determined for the individual vessel by including the 3D geometry of the coronary artery in the CFR_Pb_ determination.

CFR_Pb_ equations are valid in these forms only when the aortic pressures are the same during the resting and hyperemic states. This requirement is approximately met in the case of regular FFR measurement, where the aortic pressure usually does not change significantly after ic. adenosis provocation. The original concept defines the interval by pressure gradients [15,16]:

(1) $$\sqrt{\frac{Hyperamic\;∆p}{Resting\;∆p}}\leq CFR\leq \frac{Hyperamic\;∆p}{Resting\;∆p}\;$$

where Δ*p* is the total pressure drop along the target vessel and *CFR* is the coronary flow resistance.

### 2.4. The Calculation of Flow Separation Index

**Figure 5:**
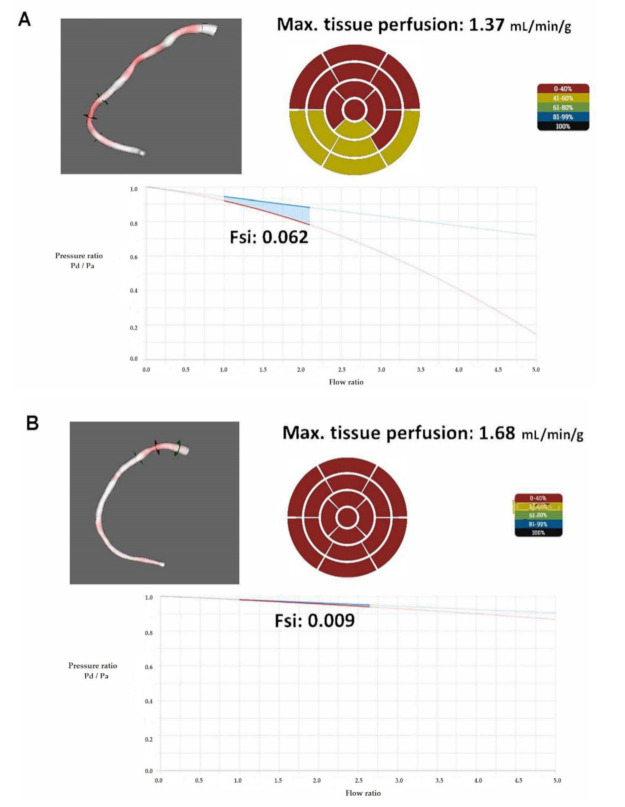
Hemodynamic changes after stent implantation in the case of patient 14. Before stent implantation (**A**) the intracoronary physiological assessment revealed a significant decrease in FFR. After stent implantation (**B**), the FFR, the CFR, and the maximal tissue perfusion increased (from 0.79 to 0.94, from 2.09 to 2.67, and from 1.37 to 1.68 mL/min/g heart muscle, respectively). The FS_i_ decreased from 0.062 to 0.009. The maximal tissue perfusion was determined from the calculated hyperemic flow divided by the supplied myocardial mass assessed on the basis of echocardiographic measurement [30] and the supplied left ventricular segments of the culprit vessel [1,31]. FS_i_: flow separation index; Max: maximum; mL: milliliter; min: minute; g: gram; Pa: pressure in the proximal reference area; Pd: pressure distal to the stenosis.

Flow separation is regarded as a disturbed flow, which is considered to be an important factor in the progression of coronary atherosclerotic plaques [27,28,29]. The hemodynamic calculation presented in Figure 4 describes the calculation and value of flow separation in the vessel-specific flow range. After calculating the CFR_p-3D_, the pressure–flow relation curve could be plotted in a diagram, which comprehensively demonstrated the flow separation pressure loss (FS_i_) (Figure 3 and Figure 4). The area of the pressure loss due to flow separation, as presented in the diagram, can be readily calculated by integrating the pressure ratio values into the patient-specific flow interval (presented as a red area in Figure 3 and Figure 4 and as a red line in Figure 5).

FS_i_ is a dimensionless parameter of the examined artery, and its value is not dependent on the size of the vessel or the absolute value of the volumetric flow. The FS_i_ value is defined proportionally to the quadratic pressure drop along a coronary lesion in an artery-specific flow range. FS_i_ is calculated with the equation:

(2) $$\mathrm{FSi}=\int_{1}^{CFR}\left(\frac{s\times Q^{2}}{P_{a}}\right)d\left(Q_{act}/Q_{rest}\right)$$

where FS_i_ is the flow separation index, *CFR* is the coronary flow reserve, *s* is the quadratic coefficient in the separation-related pressure loss term, *Q* is the volumetric flow; *Pa* is the aortic pressure, *Qact* is the actual volumetric flow, and *Qrest* is the volumetric flow registered under resting state.

### 2.5. Patient Population

Sixteen consecutive patients were enrolled into the study between 1 December 2018 and 1 April 2019. All patients had an indication for invasive coronary investigation. The main inclusion criteria of patients into our current study were single coronary lesion of left anterior descending coronary artery (LAD), left circumflex coronary artery (LCx), or right coronary artery (RCA) with intermediate stenosis. All patients were excluded who had multivessel disease, more than one lesion on the interrogated coronary artery, left main coronary artery disease, ostial stenosis, CABG in the anamnesis, or an acute indication of coronary angiography. The study was conducted according to the guidelines of the Declaration of Helsinki and approved by the Hungarian National Institute of Pharmacy and Nutrition (project identification code: OGYEI/61148/2018). Informed consent was obtained from all subjects involved in the study.

### 2.6. Statistical Analysis

Statistical evaluations were performed by using the MedCalc Statistical Software, Version 14.8.1 (MedCalc Software bvba, Ostend, Belgium). Sensitivity (SN), specificity (SP), positive predictive value (PPV), negative predictive value (NPV), and accuracy (AC) with confidence intervals (CI 95%) were calculated in a standard way. The Shapiro–Wilk test was performed to identify normal distribution. Parameter changes after stent implantation were compared by paired t-test. Spearman’s correlation analysis was carried out to examine the correlations of FS_i_ and CFR_p-3D_, as well as FFR, and a significant relationship was found between two variables if the associated *p* value was less than 0.05. The area under the curve (AUC) calculated by receiver operating characteristic (ROC) analysis was applied to determine the diagnostic power of FS_i_ to predict an FFR < 0.80.

## 3. Results

To evaluate the correlation of FS_i_ to FFR and CFR, the value was calculated for 19 coronary arteries in 16 patients. Baseline characteristics of patients are presented in Table 1. Detailed hemodynamic parameters are summarized in Table 2. In the case of the three arteries, pre- and post-PCI calculations were also performed, their detailed hemodynamic parameters and changes are featured in Table 2, and the results are indicated with A (pre-stent measurement) or B (post-stent measurement).

Hemodynamic changes of patient 14 after stent implantation are depicted in Figure 5.

**Figure 6:**
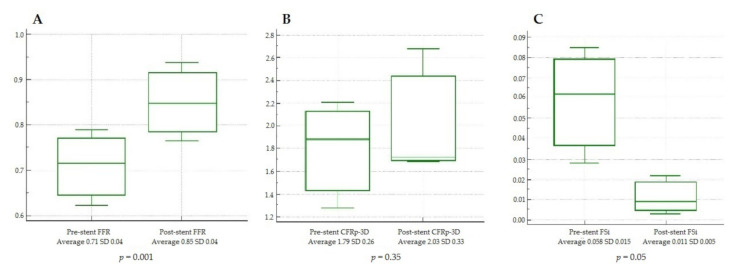
Changes of hemodynamic indices before and after stent implantation displayed as box-and-whisker plots. (**A**) Change of FFR value, (**B**) change of CFRp-3D, and (**C**) change of the FS_i_. FFR: fractional flow reserve; CFR_p-3D_: three-dimensional derived coronary flow reserve; FS_i_: flow separation index; SD: standard deviation.

The changes in the average values of FFR, CFR_p-3D_, and FS_i_ in patients 14, 15, and 16, where PCI with stent implantation was performed, are depicted in Figure 6. The changes between pre- and post-stent average values of FFR and FS_i_ differed significantly (0.71 ± 0.04 to 0.85 ± 0.04, *p* = 0.001 and 0.058 ± 0.015 to 0.011 ± 0.005, *p* = 0.05, respectively), while there was no significant difference in the CFR_p-3D_ (1.79 ± 0.26 to 2.03 ± 0.33, *p* = 0.35) in this small-sized group.

**Figure 7:**
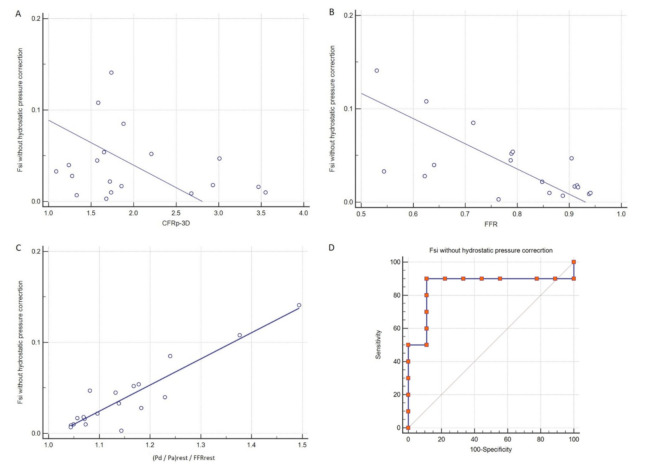
(**A**) Correlation between CFR_p-3D_ and FS_i_, (**B**) correlation between FFR and FSi, (**C**) correlation between resting (P_d_/P_a_)/FFR and FS_i_, and (**D**) ROC analysis of the FS_i_ for predicting FFR < 0.80. FFR: fractional flow reserve; CFR_p-3D_: three-dimensionally derived coronary flow reserve; FS_i_: flow separation index; P_a_: pressure in the proximal reference area; P_d_: pressure distal to the stenosis; ROC: receiver operating characteristic; rest: resting phase during pressure measurement.

In our study, no significant correlation was found between FS_i_ and CFR_p-3D_ (r = −0.23, *p* = 0.34) (Figure 7A); however, there was a significant negative correlation between FS_i_ and FFR (r = −0.66, *p* = 0.002) (Figure 7B).

An even stronger correlation was found between FS_i_ and the ratio of the resting pressures quotient (P_d_/P_a_) and FFR (r = 0.92, *p* < 0.0001) (Figure 7C). The diagnostic power of FS_i_ for predicting an FFR value of <0.80, as a gold standard prognostic factor, was tested by ROC analysis in this small-sized pilot study. An FS_i_ greater than 0.022 proved to be the cutoff value of the prediction of pathologically low FFR with a 0.856 AUC with a 95% confidence interval between 0.620 to 0.972 (Figure 7D). Positive predictive value, negative predictive value, accuracy, sensitivity, and specificity were 0.900, 0.889, 0.895, 90.00%, and 88.89%, respectively.

## 4. Discussion

In our novel interpretation, the pressure–flow relation derived from routine FFR measurement and 3D parameters provides a new index for distinction between resistance of laminar (or friction) flow and resistance of flow separation. According to the latter component, the flow separation index (FS_i_) was generated in the vessel-specific flow range (Figure 3). It seems reasonable to differentiate between benign laminar, viscous and pathologically disturbed, turbulent flows. Recent experimental results also support the idea that flow disturbance plays a causal role (lowered and multidirectional shear stress) in the progression of atherosclerotic plaques [32].

Our method for the combined determination of FFR_p-3D_ and FS_i_ is applicable for every clinically indicated invasive FFR measurement during coronary angiography after the target vessel’s 3D reconstruction.

The 3D reconstruction software, which can be either built into the X-ray equipment or operated separately on a personal computer, can provide the necessary data for the 3D reconstruction for the calculation of the flow values by using intracoronary pressure measurement results. The flow calculation itself performed by this module does not require extra calculation time and provides an online, comprehensive flow–pressure relation display. Thereby, a more comprehensive dataset can be gathered compared to the FFR measurement alone: the consequences of epicardial stenosis can be evaluated together with the complex relations of the microvascular state, while the endothelial function can also be assessed based on the correlation of CFR and FFR, where CFR/FFR will be an indicator of microvascular function. According to these values, repeated examinations during the follow-up of the patients may clearly show the effects of the treatment both on the epicardial and microvascular levels.

The microvascular level has been in the focus of clinical research lately, as accumulating data underline the importance of microvascular injury in the pathogenesis of coronavirus disease 2019 (COVID-19)–related cardiovascular complications [33]. Endothelial injury and dysfunction affecting mainly the microvascular circulation provoked by immune-mediated injury seems to be an important determinant of these complications [34,35]. In this respect, the precise examination and reification of the microvascular circulation is warranted for the assessment of sustained symptoms of severe acute respiratory syndrome coronavirus 2 (SARS-CoV-2) infection in relation to possible heart involvement. Especially in the case of SARS-CoV-2 infection–associated cardiac injury, the identification of long-term consequences is necessary by a validated method for the measurement and follow-up of microvascular function. Only an easily available diagnostic tool can open a broad field of assessment of the effect of potential treatments at the microvascular level.

In our pilot experiment, significant correlation was identified between FS_i_ and FFR. This finding is in line with previous observations that the pressure gradient during the hyperemic state of the coronary artery is linked to the increase in pathological flow separation [36]. On the other hand, FFR reflects only the hyperemic pressure gradient, and therefore, it cannot characterize the resting conditions. However, the resting pathological flow also plays an important role in the progression of the epicardial plaque for obvious reasons, exposing the low and oscillating flow shear stress in the area of the flow separation, even for longer time periods than the hyperemic one [37].

Thus, by the inclusion of basal flow characterization, FS_i_ may be a potentially better prognostic marker than only hyperemic flow–related FFR, for cardiovascular endpoints related to the progression of a coronary plaque. In this concept, the higher the value of FS_i_, the higher the risk of the progression of CHD.

## 5. Conclusions

As stated previously, the incorporation of flow parameters into descriptive coronary physiological examination is an important guide in clinical decision-making [38,39]. The present flow–pressure–velocity display provides a comprehensive summary of patient-specific pathology in CHD. The consequences of epicardial stenosis can be evaluated together with the complex relations of microvascular conditions without the requirement of Doppler wire or thermodilution procedures.

As disturbed flow is considered to be an important factor in the progression of epicardial coronary atherosclerotic plaques [20], we have differentiated benign laminar and pathologically turbulent flow resistances during routine FFR measurements. Using 3D coronary reconstruction data, the calculated pressure–flow relation provided a new, potentially prognostic information related to the FS_i_ in a vessel-specific flow range. Based on these results, a large-scale clinical follow-up trial is warranted to test the prognostic value of FS_i_. Our method may serve as a theoretical basis for prospective clinical trials investigating the natural history and effects of different CHD treatments by event-driven analysis. FS_i_ is a promising and comparable parameter to identify each coronary vessel with an atherosclerotic lesion prone to progress.

## 6. Patents

The patent of the method detailed in this paper was issued by the Hungarian Intellectual Property Office (appl. no.: PCT/HU2019/050008, applicant: University of Debrecen). Z.K. is the inventor of this patent. No other conflict of interest is declared.

## Figures and Tables

**Table 1:** Clinical characteristics of patients.

Parameter	Value
General	
Age yrs (SD)	59.56 (7.02)
Men *n* (%)	14 (87.50)
CV comorbidities	
Hypertension *n* (%)	13 (81.25)
Diabetes mellitus *n* (%)	8 (50.00)
Dyslipidemia *n* (%)	9 (56.25)
PAD *n* (%)	2 (12.50)
Cardiac conditions	
CHD *n* (%)	16 (100.00)
Previous stent implantation	10 (62.50)

Values are means ± SD or number and percentages of subjects. Yrs: years; SD: standard deviation; *n*: number; CV: cardiovascular; PAD: peripheral artery disease; CHD: atherosclerotic coronary heart disease.

**Table 2:** The 3D anatomical and hemodynamic characteristics of interrogated cases.

Case	Artery	Lesion Length 3D (mm)	Min. lumen Area 3D (mm^2^)	Diameter Stenosis (%)	Pa Rest mmHg	Pd Rest mmHg	Pa Vasodil mmHg	Pd Vasodil mmHg	FFR	CFR_p-3D_	FS_i_
1	RCA	11.3	1.62	40	73	72	67	63	0.94	3.55	0.010
2	LCx	9.0	5.62	32	108	106	96	88	0.92	3.47	0.016
3	RCA	6.6	3.56	46	104	100	100	91	0.91	1.86	0.017
4	LAD	39.3	1.69	49	93	86	94	81	0.86	1.74	0.010
5	LAD	18.7	0.44	69	93	80	80	50	0.63	1.58	0.108
6	LAD	27.1	1.17	64	89	70	75	48	0.64	1.24	0.040
7	LAD	16.5	2.79	44	117	109	91	72	0.79	1.65	0.054
8	LAD	15.6	2.32	47	95	88	89	79	0.89	1.33	0.007
9	RCA	11.7	2.23	70	91	89	94	85	0.90	3.01	0.047
10	LAD	53.8	0.95	70	96	76	100	53	0.53	1.74	0.141
11	LCx	34.0	0.78	77	105	65	103	56	0.54	1.09	0.033
12	LCx	8.2	1.98	39	90	88	82	75	0.91	2.93	0.018
13	LAD	19.7	2.11	52	110	98	108	85	0.79	1.57	0.045
14A	RCA	12.2	1.00	54	101	93	104	82	0.79	2.21	0.062
14B	RCA	14.4 *	2.38 *	37 *	97	95	97	91	0.94	2.68	0.009
15A	LAD	36.1	1.41	60	115	102	116	83	0.72	1.88	0.085
15B	LAD	18.1 *	2.75 *	42 *	113	105	105	89	0.85	1.72	0.022
16A	LAD	50.7	0.80	57	87	64	82	51	0.62	1.28	0.028
16B	LAD	8.4 *	4.78 *	26 *	79	69	72	55	0.76	1.68	0.003
Mean	na.	21.65	2.13	51.32	97.68	87.11	92.37	72.47	0.79	2.01	0.040
SD	na.	11.69	0.99	11.70	9.63	12.09	10.63	13.87	0.11	0.61	0.028

A: pre-stent measurement of patient if percutaneous coronary intervention is performed; B: post-stent measurement of same patient after percutaneous coronary intervention; RCA: right coronary artery; LCx: left circumflex coronary artery; LAD: left anterior descending coronary artery; mm: millimeter; min: minimum; mm^2^: square millimeter, FFR: fractional flow reserve, CFRp-3D: three-dimensional derived coronary flow reserve; FSi: flow separation index; 3D: three dimensional; Pa: pressure in the proximal reference area; Pd: pressure distal to the stenosis; mmHg: millimeter of mercury; vasodil: under maximal vasodilatation; rest: under resting condition; na.: not applicable; SD: standard deviation; * 3D parameters after stent implantation were detected outside the stented segments automatically by the 3D quantitative coronary angiography software.

## Data Availability

The data presented in this study are available on request from the corresponding author. The data are not publicly available due to privacy.
